# Complete genome sequence of the motile actinomycete *Actinoplanes missouriensis* 431^T^ (= NBRC 102363^T^)

**DOI:** 10.4056/sigs.3196539

**Published:** 2012-12-18

**Authors:** Hideki Yamamura, Yasuo Ohnishi, Jun Ishikawa, Natsuko Ichikawa, Haruo Ikeda, Mitsuo Sekine, Takeshi Harada, Sueharu Horinouchi, Misa Otoguro, Tomohiko Tamura, Ken-ichiro Suzuki, Yasutaka Hoshino, Akira Arisawa, Youji Nakagawa, Nobuyuki Fujita, Masayuki Hayakawa

**Affiliations:** 1 Division of Applied Biological Sciences, Interdisciplinary Graduate School of Medicine and Engineering, University of Yamanashi, Yamanashi, Japan; 2 Department of Biotechnology, Graduate School of Agricultural and Life Sciences, The University of Tokyo, Tokyo, Japan; 3 Department of Bioactive Molecules, National Institute of Infectious Diseases, Tokyo, Japan; 4 Biological Resource Center, National Institute of Technology and Evaluation, Tokyo, Japan; 5 Kitasato Institute for Life Sciences, Kitasato University, Kanagawa, Japan; 6 Biological Resource Center, National Institute of Technology and Evaluation, Chiba, Japan; 7 Bioresource Laboratories, MicroBiopharm Japan Co., Ltd., Shizuoka, Japan

**Keywords:** motile actinomycetes, sporangia, zoospores, motile spores, flagellation, aerobic, Gram-positive, *Micromonosporaceae*, *Actinoplanes*, *A. missouriensis*

## Abstract

*Actinoplanes missouriensis* Couch 1963 is a well-characterized member of the genus *Actinoplanes*, which is of morphological interest because its members typically produce sporangia containing motile spores. The sporangiospores are motile by means of flagella and exhibit chemotactic properties. It is of further interest that members of *Actinoplanes* are prolific sources of novel antibiotics, enzymes, and other bioactive compounds. Here, we describe the features of *A. missouriensis* 431^T^, together with the complete genome sequence and annotation. The 8,773,466 bp genome contains 8,125 protein-coding and 79 RNA genes.

## Introduction

Strain 431^T^ [= NBRC 102363^T^ = DSM 43046^T^ = ATCC 14538^T^ and other culture collections] is the type strain of the species *Actinoplanes missouriensis* [1,2], which is a well-characterized member of the genus *Actinoplanes* [3]. The genus is of morphological interest because its members typically produce spherical, subspherical, cylindrical, or very irregular sporangia arising from vegetative mycelia [1,4]. Sporangiospores, which are released following the immersion of sporangia in water, are motile by means of polar or peritrichous flagella [5] and display a positive chemotactic response to a number of amino acids, aromatic compounds, sugars and inorganic ions [6,7]. Recently, the flagellin gene of various *Actinoplanes* strains was successfully amplified and sequenced to reveal the evolutionary relationships between flagellar genes [8]. As members of the genus *Actinoplanes*, including *A. missouriensis*, produce a variety of antibiotics, enzymes and other bioactive compounds [9-12], the genomes of *Actinoplanes* species are potentially useful genetic resources for discovering secondary metabolites and enzymes. The genome analysis of the acarbose producer *Actinoplanes sp.* SE50/110 was most recently reported [13].

Here, we present a summary classification and a set of features for *A. missouriensis* strain 431^T^, together with a description of the complete genome sequencing and annotation.

## Classification and features

*A. missouriensis* strain 431^T^ was originally isolated from barnyard soil near Hamilton, Missouri, USA using the pollen-baiting technique and was first described by Couch in 1963 [1]. Couch also isolated additional strains of *A. missouriensis* from ten soil collections obtained from the Mississippi Valley to the West Coast. The range of 16S rRNA gene sequence similarities between strain 431^T^ (= NBRC 102363^T^) (AB711914) and valid members of the genus *Actinoplanes* was 95.7–97.9%. The highest sequence similarities were to *Actinoplanes utahensis* NBRC 13244^T^.

**Figure 1:**
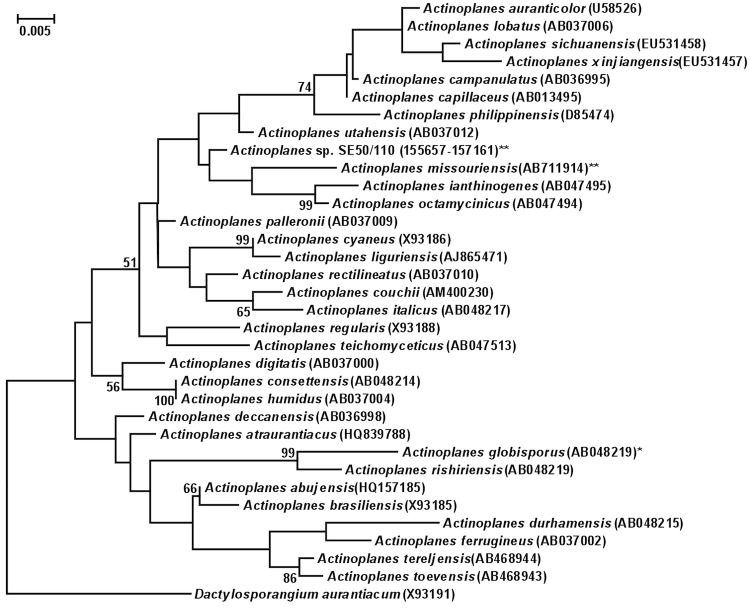
Phylogenetic tree highlighting the position of *A. missouriensis* 431^T^ relative to *Actinoplanes sp.* strain SE50/110 and other type strains within the genus *Actinoplanes*. The tree was inferred from 1,351 aligned characters [14,15] of the 16S rRNA gene sequence under the maximum likelihood criterion [16] and rooted with the type strain of the neighboring genus *Dactylosporangium*. Only bootstrap values above 50% are shown (1,000 resamplings) at branching points. Lineages with type strain genome sequencing projects registered in GOLD [17] are labeled with an asterisk, those also listed as 'Complete and Published' with two asterisks.

Figure 1 shows the phylogenetic neighborhood of *A. missouriensis* 431^T^ in a 16S rRNA gene-based tree. The sequences of five of the six 16S rRNA gene copies in the genome of *A. missouriensis* 431^T^ are identical (one sequence differed by 4 nucleotides), but differ by 55 nucleotides (3.8%) from the previously published 16S rRNA gene sequence of NBRC 13243^T^ (AB037008). The differences between the genome data and the reported 16S rRNA gene sequence are likely due to sequencing errors in the previously reported sequence data. The 16S rRNA sequence of strain 431^T^ did not match significantly with any 16S rRNA gene sequences from environmental genomic samples and surveys available at the NCBI BLAST server (March 2012).

**Figure 2:**
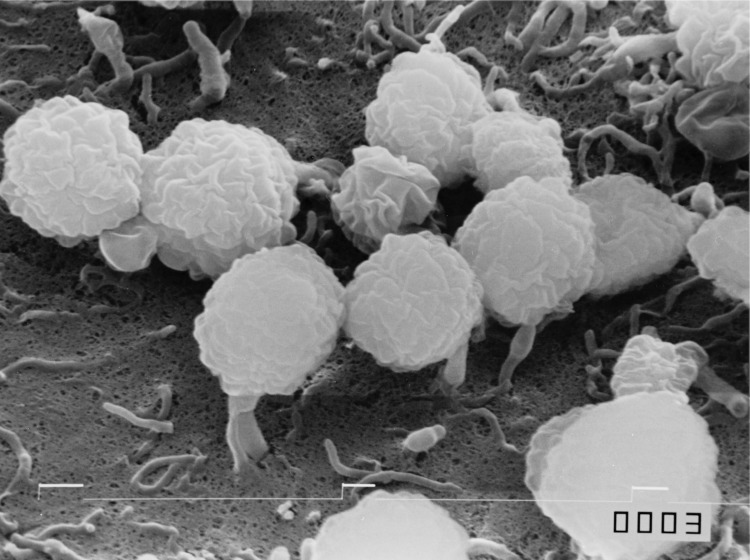
Scanning electron micrograph of *A. missouriensis* 431^T^. Bar interval, 10 µm.

The color of the substrate mycelia of strain 431^T^ is ochraceous salmon on Czapek agar. The strain does not form aerial mycelium. On Czapek agar, the strain produces a soluble pale lavender pigment [1]. Optimum growth occurs at 28°C. Strain 431^T^ produces globose spores (1-1.2 µm) arranged in irregular coils within a terminal sporangium. Sporangia are globose to subglobose in shape and 6 to 14 µm in diameter [Figure 2] [1].

Sporangia release motile spores, called zoospores, after unwrapping of the membranous sheath covering sporangia (Table 1) [5]. Released zoospores have chemotactic properties for several substrate types, including sugars, amino acids, aromatic compounds and mineral ions [6,7]. Flagella of the zoospores consist of a 44-kDa flagellar protein (FliC) [5] encoded by the *fliC* gene, which has been applied as a molecular marker in a taxonomic study of the genus *Actinoplanes* [8]. Strain 431^T^ utilizes D-xylose, L-arabinose, D-glucose, D-fructose, D-mannose, L-rhamnose, D-mannitol, sucrose, mannose, dextrin, D-galactose, D-lactose, methyl β-D-glucoside and L-rhamnose (1%, w/v), but not *myo*-inositol, raffinose, cellulose, adonitol, D-arabinose, D-melezitose, methyl α-D-glucoside, D-ribose or D-sorbitol (1%, w/v) [4,29]. Strain 431^T^ also utilizes *p*-hydroxybenzoic acid, sodium acetate and sodium fumarate (0.1%, w/v), but not *m*-hydroxybenzoic acid, sodium succinate, syringic acid, or vanillin (0.1%, w/v) [4,29]. Strain 431^T^ degrades chitin, DNA, elastin, lecithin, RNA and tyrosine (26), and also actively decomposes natural rubber [32] and flavonoids, such as quercetin, rutin and hesperidin [33]. Strain 431^T^ produces glucose isomerase (xylose isomerase) with a molecular weight of approximately 8,000 daltons [34] and also produces 6-alkyl-4-*O*-dihydrogeranyl-2-methoxyhydroquinones as novel phenolic lipids [35]. Antimicrobial activity against *Streptomyces murinus* ISP 5091 is positive, but is negative against *Aspergillus niger* LIV 131, *Bacillus subtilis* NCIB 3610 and *Staphylococcus aureus* NCTC 8532 [29]. Additional physiological and drug susceptibility data are described in detail elsewhere [29].

**Table 1:** Classification and general features of *A. missouriensis* 431^T^ based on the MIGS recommendations [18]

**MIGS ID**	**Property**	**Term**	**Evidence code**
		Domain *Bacteria*	TAS [19]
		Phylum *Actinobacteria*	TAS [20]
		Class *Actinobacteria*	TAS [21]
	Current classification	Order *Actinomycetales*	TAS [2,21-24]
		Family *Micromonosporaceae*	TAS [2,21,24-26]
		Genus *Actinoplanes*	TAS [2,3,27,28]
		Species *Actinoplanes missouriensis*	TAS [1,2]
		Type strain 431	TAS [1,2]
	Gram stain	Not tested, likely positive	NAS
	Cell shape	Produces sporangium	TAS [1]
	Motility	Motile	TAS [1]
	Sporulation	Motile spore	TAS [1]
	Temperature range	Mesophile, temperature range not determined	TAS [29,30]
	Optimum temperature	28°C	TAS [29,30]
	Salinity	2% NaCl	TAS [29]
MIGS-22	Oxygen requirement	Aerobe	NAS
	Carbon source	Several (refer to text)	TAS [29]
	Energy source	Carbohydrates	TAS [29]
MIGS-6	Habitat	Soil	TAS [1]
MIGS-15	Biotic relationship	Free-living	NAS
MIGS-14	Pathogenicity	None	NAS
	Biosafety level	1	NAS
	Isolation	Barnyard soil	TAS [1]
MIGS-4	Geographic location	Hamilton, Missouri, USA	TAS [1]
MIGS-5	Sample collection time	1963 or before	TAS [1]
MIGS-4.1	Latitude	Not reported	
MIGS-4.2	Longitude	Not Reported	
MIGS-4.3	Depth	Not reported	
MIGS-4.4	Altitude	Not reported	

Evidence codes - IDA: Inferred from Direct Assay (first time in publication); TAS: Traceable Author Statement (i.e., a direct report exists in the literature); NAS: Non-traceable Author Statement (i.e., not directly observed for the living, isolated sample, but based on a generally accepted property for the species, or anecdotal evidence). These evidence codes are from the Gene Ontology project [31]. If the evidence code is IDA, then the property was directly observed by one of the authors or an expert mentioned in the acknowledgements.

## Chemotaxonomy

The diagnostic amino acids of strain 431^T^ are *meso*- and hydroxydiaminopimelic acids, and the characteristic whole-cell sugars are xylose and arabinose [29,36]. The peptidoglycan of strain 431^T^ contains glycosylated muramic acid residues [28,37], and the major phospholipids are phosphatidylethanolamine, diphosphatidylglycerol, phosphatidylglycerol, phosphatidylinositol and phosphatidylinositol mannoside [28]. Major fatty acids (relative ratio %) are iso-C_16:0_ (18.3) and C_19:0_ (26.4) [28,29]. The predominant menaquinone is MK-9(H_4_) (>50%), and the minor menaquinones are MK-9(H_6_) and MK-10(H_4_) [28,29].

## Genome sequencing information

### Genome project history

A consortium consisting of universities, research institutions and private companies in Japan was organized to accelerate cooperative research and development utilizing genome sequences of actinomycetes. The consortium successfully accomplished the genome projects of *Kitasatospora setae* NBRC 14216^T^ [38] and *A. missouriensis* 431^T^ (= NBRC 102363^T^), as reported here. The complete genome sequences of these strains have been deposited in the INSDC database and are also available from the DOGAN genome database [39]. A summary of the genome sequencing project information of *A. missouriensis* is shown in Table 2.

**Table 2:** Genome sequencing project information

**MIGS ID**	**Property**	**Term**
MIGS-31	Finishing quality	Complete
MIGS-28	Libraries used	Three Sanger libraries: 1.5- and 6-kb pUC118, and 36-kb fosmid pCC1Fos
MIGS-29	Sequencing platform	Sanger ABI 3730xl
MIGS-31.2	Fold coverage	8.7 ×
MIGS-30	Assemblers	Phrap
MIGS-32	Gene calling method	Glimmer3, tRNAscan-SE 1.23
	Genome database release	DOGAN [39]
	Genbank ID	AP012319
	Genbank date of release	2012/03/31
	GOLD ID	Gc02182
	Project relevance	Biotechnological

### Growth conditions and DNA isolation

*A. missouriensis* strain 431^T^ (= NBRC 102363^T^) was grown in NBRC 231 medium (Maltose-Bennett’s Agar) at 28°C. DNA was isolated from 3.76 g of mycelial paste using the CTAB method and fragmented with the HydroShear device (Genomic Solutions), as recommended by the manufacturer.

### Genome sequencing and assembly

The genome sequence of *A. missouriensis* 431^T^ was determined using a whole-genome shotgun sequencing approach together with the Sanger method. DNA shotgun libraries with average insert sizes of 1.6 and 6 kbp were constructed in pUC118, and a library with an average insert size of 36 kbp was constructed in pCC1FOS (Epicentre). A total of 96,384 reads were trimmed at a threshold quality value of 20 and assembled using the Phrap and CONSED assembly tools [40,41]. A total of 141 gaps (18,532 bp in total) were closed by sequencing PCR products, and 236 low-quality regions were re-sequenced by primer walking. In the final assembly step, we confirmed that each base of the genome was sequenced from multiple clones in either both or a single direction and had Phrap quality scores of >70 and >40, respectively. For validation of the contig alignment, the Argus Optical Mapping System (OpGen, Inc.) was used.

### Genome annotation

Gene prediction was performed using Glimmer3 [42] and tRNA-scanSE [43], followed by manual inspection of each translation start site using the Frameplot program [44]. Similarity search results against the NCBI nr and Pfam databases were used for functional prediction. Manual functional annotation was performed within an in-house platform developed by J.I. (Unpublished).

## Genome properties

**Figure 3:**
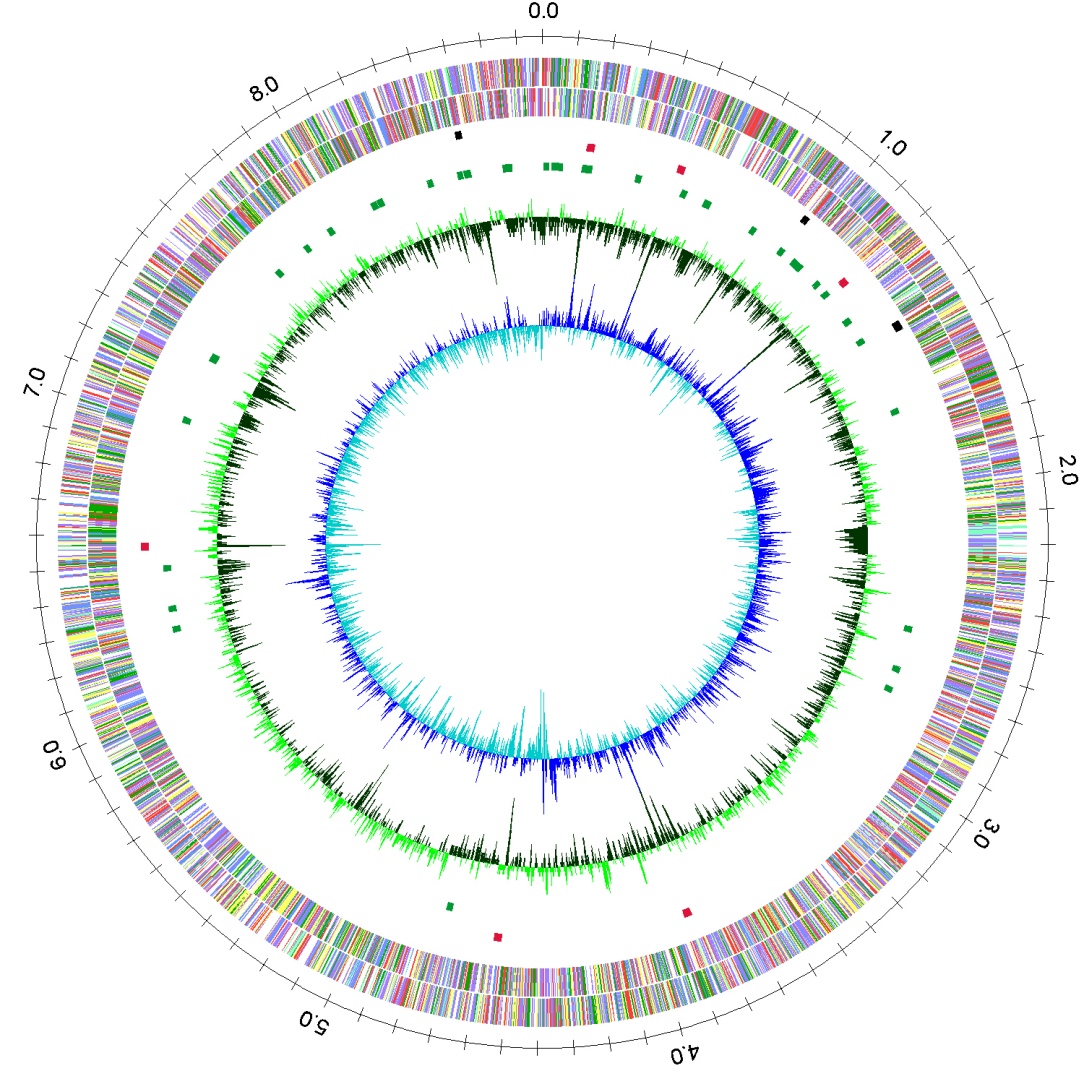
Graphical circular map of the strain 431^T^ genome. From outside to the center: genes on forward strand (color by COG categories), Genes on reverse strand (color by COG categories), RNA genes (tRNA genes, green; rRNA genes, red; other RNA gene types, black), GC content, GC skew.

The genome of strain 431^T^ consists of one circular chromosome with a length of 8,773,466 bp and a G+C content of 70.8% ( Table 3 and Figure 3). *A. missouriensis* DSM 43046^T^ was reported to contain a linear plasmid, pAM1 [33]; however, the corresponding plasmid was not found in strain 431^T^ (= NBRC 102363^T^) by CHEF electrophoresis analysis. It is possible that the linear plasmid was cured from strain 431^T^ by repeated subculturing in the laboratory. In comparison to other members of the family *Micromonosporaceae*, such as *Salinispora* and *Micromonospora*, *A. missouriensis* has a larger genome size. Of the total of 8,204 predicted genes, 8,125 were protein-coding genes and 79 were RNA genes. More than half of the protein-coding genes (4,539, 55.9%) were assigned a putative function, while the remaining predicted genes were annotated as hypothetical proteins. The distribution of genes into COGs functional categories is presented in Table 4.

**Table 3:** Genome statistics

**Attribute**	**Value**	**% of total^a^**
Genome size (bp)	8,773,466	100.00%
Coding region (bp)	7,964,669	90.78%
G+C content (bp)	6,213,639	70.82%
Total genes	8,204	n/a
RNA genes	79	n/a
rRNA operon	6	n/a
tRNA (44 species)	58	n/a
tmRNA	1	n/a
4.5S RNA	1	n/a
*rnpB* (RNase P component)	1	n/a
Protein-coding genes	8,125	100.00%
Genes in paralog clusters	3,123	38.44%
Genes assigned to COGs	5,171	63.64%
Genes assigned Pfam domains	5,463	67.24%
Genes with signal peptides^b^	799	9.83%
Genes with transmembrane helices	2,122	26.12%

a) The total is based on either the size of the genome in base pairs or the total number of protein-coding genes in the annotated genome.

b) Predicted by SignalP 4.0.

n/a, not available

**Table 4:** Number of genes associated with the 25 general COG functional categories

**Code**	**Value**	**%age**^a^	**Description**
J	199	2.4	Translation, ribosomal structure and biogenesis
A	1	0	RNA processing and modification
K	831	10.2	Transcription
L	234	2.9	Replication, recombination and repair
B	1	0	Chromatin structure and dynamics
D	42	0.5	Cell cycle control, mitosis and meiosis
Y	0	0	Nuclear structure
V	143	1.8	Defense mechanisms
T	528	6.5	Signal transduction mechanisms
M	240	3.0	Cell wall/membrane biogenesis
N	80	1.0	Cell motility
Z	1	0	Cytoskeleton
W	0	0	Extracellular structures
U	52	0.6	Intracellular trafficking and secretion
O	156	1.9	Posttranslational modification, protein turnover, chaperones
C	360	4.4	Energy production and conversion
G	579	7.1	Carbohydrate transport and metabolism
E	524	6.4	Amino acid transport and metabolism
F	91	1.1	Nucleotide transport and metabolism
H	199	2.4	Coenzyme transport and metabolism
I	286	3.5	Lipid transport and metabolism
P	326	4.0	Inorganic ion transport and metabolism
Q	236	2.9	Secondary metabolites biosynthesis, transport and catabolism
R	976	12.0	General function prediction only
S	387	4.8	Function unknown
-	2,954	36.4	Not in COGs

a) The total is based on the total number of protein-coding genes in the annotated genome.
